# Comparative Study of Titanium-Doped and Titanium–Silver Co-Doped Diamond-Like Carbon Films

**DOI:** 10.1021/acsomega.5c10368

**Published:** 2025-12-24

**Authors:** Oskars Platnieks, Liutauras Marcinauskas, Hassan Zhairabany, Anatolijs Sarakovskis, Edgars Vanags, Sergejs Gaidukovs, Hesam Khaksar, Enrico Gnecco

**Affiliations:** † Department of Physics, 70309 Kaunas University of Technology, Kaunas, Studentų g. 50, LT-51369, Lithuania; ‡ 487441 University of Latvia, Institute of Solid State Physics, Riga LV-1063, Latvia; § Faculty of Natural Sciences and Technology, Institute of Chemistry and Chemical Technology, 87254 Riga Technical University, P. Valdena Str. 3, Riga LV-1048, Latvia; ∥ Marian Smoluchowski Institute of Physics, 37799 Jagiellonian University, Krakow 30-348, Poland

## Abstract

Hydrogen-free diamond-like carbon (DLC) films were deposited by magnetron sputtering and doped with titanium (Ti-DLC) and codoped with titanium and silver (Ti/Ag-DLC, 80/20 at. % TiAg target). Ti loadings of 0.3–1.8 at. % produced only modest roughness changes (*R*_q_ ≈ 1.8–2.3 nm) and a slight increase in *I*_D_/*I*_G_ and sp^2^/sp^3^ ratios, though the D-band down-shifted markedly. Ti/Ag codoped DLC films contained 1.0–6.9 at. % total metal, while the surface was enriched in Ag according to X-ray photoelectron spectroscopy. Except for the highest doped film, Ti/Ag-DLC showed lower graphitization than the Ti-DLC films prepared under identical conditions. *R*_q_ increased to 3.9 nm for the Ti/Ag-DLC films, reaching the highest value at the lowest Ti/Ag content. The presence of Ag also diminished surface oxidation and reduced oxygen concentration at low doping levels. Ti doping and Ti/Ag codoping of DLC films reduced the coefficient of friction by up to 2-fold when normal loads of 1–10 nN were used. Nanoindentation tests revealed that both Ti-DLC and Ti/Ag-DLC films show their greatest hardness loss at the lowest dopant concentrations. Water contact angles for Ti-DLC films changed nonmonotonically but became slightly less hydrophilic (∼69°) compared to undoped DLC. Ti/Ag-DLC films first became more wettable (59.9°) and then recovered to 68.5° as metal content increased. OWRK calculations showed a dopant-induced decline in total surface free energy, which was driven by reductions in dispersive components. Collectively, these data indicate that the Ti/Ag codoping offers a tunable balance of hardness, roughness, and wettability, combining the benefits of Ti with the advantages of Ag when applied at low to moderate concentrations.

## Graphical abstract

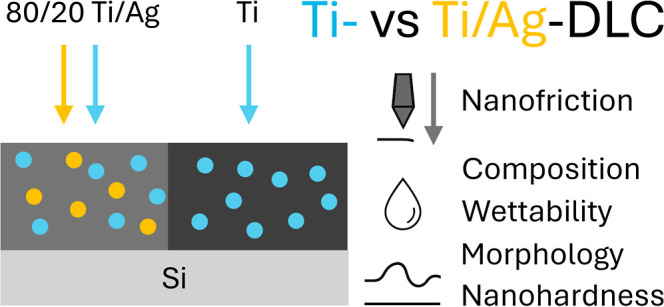

## 1 Introduction

The addition of metallic dopants is one of the most effective strategies for tailoring the properties of diamond-like carbon (DLC) films.^1^ While many studies have characterized metal-doped DLC under conventional sliding,^2–4^ the nanoscale friction behavior of such films remains far less explored.^5,6^ Studying friction behavior at the nanoscale is crucial because the governing mechanisms can differ significantly from those at the microscale or macroscale.^7^ In a single contact (such as an atomic force microscopy (AFM) tip sliding on a film), friction is dominated by adhesive forces and interfacial shear at the tip–sample junction.^8^ Moreover, advanced technologies, including micro-/nanoelectromechanical systems, magnetic storage devices, and biomedical implants, operate under light loads and small contact areas, where nanoscale tribological response directly governs long-term performance.^8^

Selecting the appropriate metallic dopants can be challenging, but they are generally classified into two functional categories in DLC systems. Hardening dopants such as Ti, W, or Ni tend to form carbides or oxides, which anchor the carbon network, enhance adhesion, and raise wear and corrosion resistance.^9,10^ By contrast, softening dopants such as Ag, Cu, or Al segregate within the matrix, reducing internal stress and friction.^11–13^ The interplay between these two classes forms the basis for multidopant strategies. Titanium incorporation is known to lower internal stress and strengthen adhesion, typically without a significant compromise in hardness.^14,15^ Ti-DLC coatings also show lower wear rates and can maintain a low friction coefficient under dry sliding.^16,17^ At the same time, surface oxidation of Ti creates protective TiO_2_ layers that improve resistance to corrosive environments.^18^ Patnaik et al. reported that an Ag-DLC film exhibited a four order-of-magnitude reduction in wear rate compared to an undoped DLC, accompanied by a lower friction coefficient, due to the emergence of a silver-rich lubricating film during sliding.^19^ Chemically, Ag inclusion has a relatively neutral or slightly beneficial effect on the corrosion behavior of DLC.^20,21^ Ag-DLC surfaces often become more hydrophilic^21^ than pure DLC, which is attributed to the oxide formation, but the opposite can be true due to increased surface roughness.^22^

Multicomponent DLC with aluminum and titanium has been shown to produce films with exceptionally low internal stress while retaining high hardness.^15^ Guo et al. found that Ti/Al codoped DLC uniquely combines low compressive stress with high hardness.^15^ Al relaxes stress by interrupting the carbon matrix, whereas Ti reinforces the structure and sustains hardness. The films further display excellent tribological performance and adhesion. In a series of studies, Xu et al. found that varying the Al/Ti ratio tunes the properties: higher Al (relative to Ti) minimizes stress (down to <0.5 GPa, far less than typical DLC) at some cost to hardness, while higher Ti enhances hardness at the expense of a modest stress increase.^23,24^ Adding Ti to DLC codoped with Cu and Ce triggers TiC formation, sealing corrosion pathways and markedly enhancing corrosion resistance.^25^ Ti therefore safeguards the carbon matrix, even in the presence of other metallic dopants, offsetting barrier losses caused by more electropositive metals. Researchers have also investigated combinations like W/Al and Cr/Al dopants in DLC.^26^ Residual stress can be significantly reduced by these combinations without compromising the mechanical properties. The consistent theme is that dual or multidopants can be tuned to optimize specific properties. These comparisons highlight that the codoping is part of a larger trend. Thus, Ti/Ag stands out as a promising pair, especially for applications requiring a mix of mechanical robustness, good adhesion, self-lubrication, and bioactivity.

In the context of the nanoscale friction of DLC systems, the structural nature of the surface plays a dominant role. For hydrogen-free DLC, the outermost layer of the film is reported to be enriched in sp^2^-bonded carbon clusters.^27^ A combination of AFM-based nanowear tests and conducting AFM, performed by simultaneously measuring the surface topography and electrical conductivity, reveals that the hydrogen-free DLC films are covered by a thin (1.5–2.0 nm) graphite-like surface layer.^28^ Consequently, the nanoscale friction behavior of hydrogen-free DLC films is strongly governed by the stability of this sp^2^-rich surface layer, which acts as the primary zone controlling interfacial shear.^29,30^ Kolodziejczyk et al. showed that introducing silver into DLC at contents of 4.5, 8.4, and 15.19 at. % progressively raises nanoscale friction, with the coefficient of friction increasing from about 0.17 for pure DLC to 0.21 for the highest Ag loading when measured with a DLC-coated tip.^5^ The authors also observed that the rise in friction is accompanied by an increase in surface roughness, driven by Ag-rich nanoclusters that become more pronounced as silver content increases, ultimately leading to higher wear compared to undoped DLC. Therefore, when introducing metal dopants into DLC, it is essential to examine how metal clustering at the surface may disrupt the formation of the native sp^2^-rich tribolayer or lead to the creation of metal-oxide bonds, suggesting that careful control and limitation of metal content is crucial to preserving the exceptional nanofriction performance of DLC films. To the best of our knowledge, apart from our own previous work, no studies have investigated the nanoscale friction coefficient of codoped DLC films.^6,31^

This work presents a systematic comparison of Ti/Ag codoped and Ti-doped diamond-like carbon (DLC) films deposited under identical magnetron-sputtering conditions. Ti, a hardening dopant, and Ag, a softening dopant, were selected to explore potential synergistic effects on the film structure and performance. Only a couple of studies have addressed on the Ti/Ag codoped DLC films: one investigated hydrogenated DLC (a-C/H) coatings,^32^ fundamentally different from the non-hydrogenated films considered here, while our earlier work^31^ focused on higher Ti/Ag dopant levels in DLC films (up to 10.7 at. %) using a 50/50 at. % Ti/Ag target. In contrast, the present study employs an 80/20 at. % TiAg target and achieves total metal loadings of 1.0–6.9 at. % (X-ray photoelectron spectroscopy (XPS)), focusing on the retention of low nanofriction. By evaluating how variations in Ti and Ti/Ag content influence the surface morphology, sp^2^/sp^3^ ratio, chemistry, wettability, mechanical response, and nanoscale friction behavior, we identify the specific effects introduced by Ag as a codopant in DLC films.

## 2 Experimental Section

### 2.1 Film Deposition

Non-hydrogenated DLC films were prepared by direct current magnetron sputtering onto single-crystal Si (100) wafers. Three high-purity (99.9%) targets (Testbourne Ltd.) supplied the vapor flux: graphite, metallic titanium, and a titanium–silver alloy (80/20 at. %). Full details of the general sputtering configuration can be found in an earlier report.^33^

During deposition, the chamber was evacuated to ∼10^–2^ Pa before Ar was introduced as the working gas (2–3 Pa). A brief Ar plasma cleaning step preceded growth. The carbon flux was delivered from the graphite source at 1.5 A, while the Ti or TiAg cathode operated at 0.25 A. Substrates were placed 6 cm away and subjected to a slow oscillatory motion to promote lateral uniformity. Temperature stabilized near 225 °C within several minutes of ignition. A mechanical shutter partially covered the metallic cathode to restrict its flux, thereby tuning the effective dopant concentration. Based on our previous measurements on this deposition setup, the films typically fall within a thickness range of 150–210 nm, with the specific conditions used in this work producing films of approximately 180 nm.^33^

### 2.2 Testing Methods

Surface topography and nanotribology were investigated by atomic force microscopy (DriveAFM, Nanosurf) using probes (PPP-LFMR and PPP-NCHR; Nanosensors) under ambient conditions (20 °C). A PPP-LFMR probe was operated in the lateral force mode. The normal load was stepped between 1 and 10 nN (spring constant: 0.4096 N m^–1^). In the tapping mode, three distinct 5 × 5 μm^2^ regions per sample were used with the scan resolution set at 256 points per line, with a total of four lines scanned using a trace and retrace approach (each line within 100 ms). In Lateral Force Microscopy (LFM), two lateral loops per point were performed, corresponding to both trace and retrace passes. The friction force calibration followed the methodology outlined in the previous work.^31^ For high-resolution surface imaging, a PPP-NCHR probe was used in the tapping mode. This probe operated at a resonance frequency of 348 kHz with a spring constant of 35.19 N m^–1^.

Elemental composition of the coatings was assessed using a Bruker Quad 5040 energy-dispersive X-ray (EDS) spectrometer. The EDS detector collected characteristic X-ray emission lines, allowing quantification of the C, O, Ti, and Ag contents within the DLC films. Measurements were taken from three surface locations to minimize local variability and improve the statistical reliability.

X-ray photoelectron spectroscopy (XPS) was performed by using a Thermo Fisher ESCALAB Xi+ system with a monochromatic Al Kα source. Measurement conditions and calibration procedures followed our previous report,^22^ with only minor adjustments. Briefly, high-resolution spectra were acquired at 20 eV pass energy and survey scans at 150 eV, and a low-energy Ar cluster gun was applied for surface cleaning.

Mechanical characterization employed a nanoindenter (Agilent G200) operated in the continuous stiffness measurement mode with the dynamic loading set to a harmonic displacement amplitude of 2 nm at a driving frequency of 45 Hz. The nominal strain rate during indentation was maintained at 0.05 s^–1^. For data evaluation, the substrate material was assigned a modulus of 152 GPa, while Poisson’s ratios of 0.27 (substrate) and 0.20 (film) were used in the calculations. Indentation depth was limited to 1 μm to minimize substrate influence, with drift corrections applied automatically. At least 25 indents were made per sample.

A Theta Lite optical tensiometer (Attension) was operated using the static sessile drop method to determine contact angle values. At least 6 droplets were deposited for each sample and liquid, and values were recorded after 5 s. Distilled water (W), diiodomethane (DM), and ethylene glycol (EG) were deposited under ambient conditions. The surface energy was calculated using Attension software, following the Owens-–Wendt–Rabel–Kaelble (OWRK) method with the following parameters: W­(γ_tot_ = 72.8, γ_d_ = 21.8, γ^+^ = 25.5, and γ^–^ = 25.5), DM­(γ_tot_ = 50.8, γ_d_ = 50.8, γ^+^ = 0, and γ^–^ = 0), and EG­(γ_tot_ = 48.0, γ_d_ = 29.0, γ^+^ = 3.00, and γ^–^ = 30.1).

Micro-Raman spectra were acquired with a Renishaw inVia spectrometer equipped with a 532 nm solid-state excitation laser. For each coating, three distinct surface regions were probed, and the final spectrum was obtained by averaging five successive accumulations. The excitation power at the sample surface was limited to 0.5 mW with an individual integration time of 10 s per accumulation. Scattered light in the Stokes region was dispersed using a grating containing 2400 grooves mm^–1^ and recorded by a Peltier-cooled CCD detector (1024 × 256 pixel resolution). Instrument calibration was performed against the characteristic silicon reference line, and spectral deconvolution of the D and G bands was achieved by using Gaussian fitting.

## 3 Results and Discussion

### 3.1 Composition

As illustrated in Figure 1, the EDS mapping of the Ti_2 and TiAg_2 films offers information about the spatial distributions of O, Ti, C, and Ag within the DLC films. While all elements are detectable throughout the mapped areas, the dopant metals exhibit a heterogeneous distribution, with Ti and Ag appearing in small-sized randomly distributed clusters. The Ti and Ag metals do not form large (higher than 1 μm) clusters or segregation areas of metals in the Ti-DLC and Ti/Ag-DLC films. This indicates that the sputtering methodology used allowed for an even distribution of metal dopants throughout the entire volume of DLC films.

**Figure 1:**
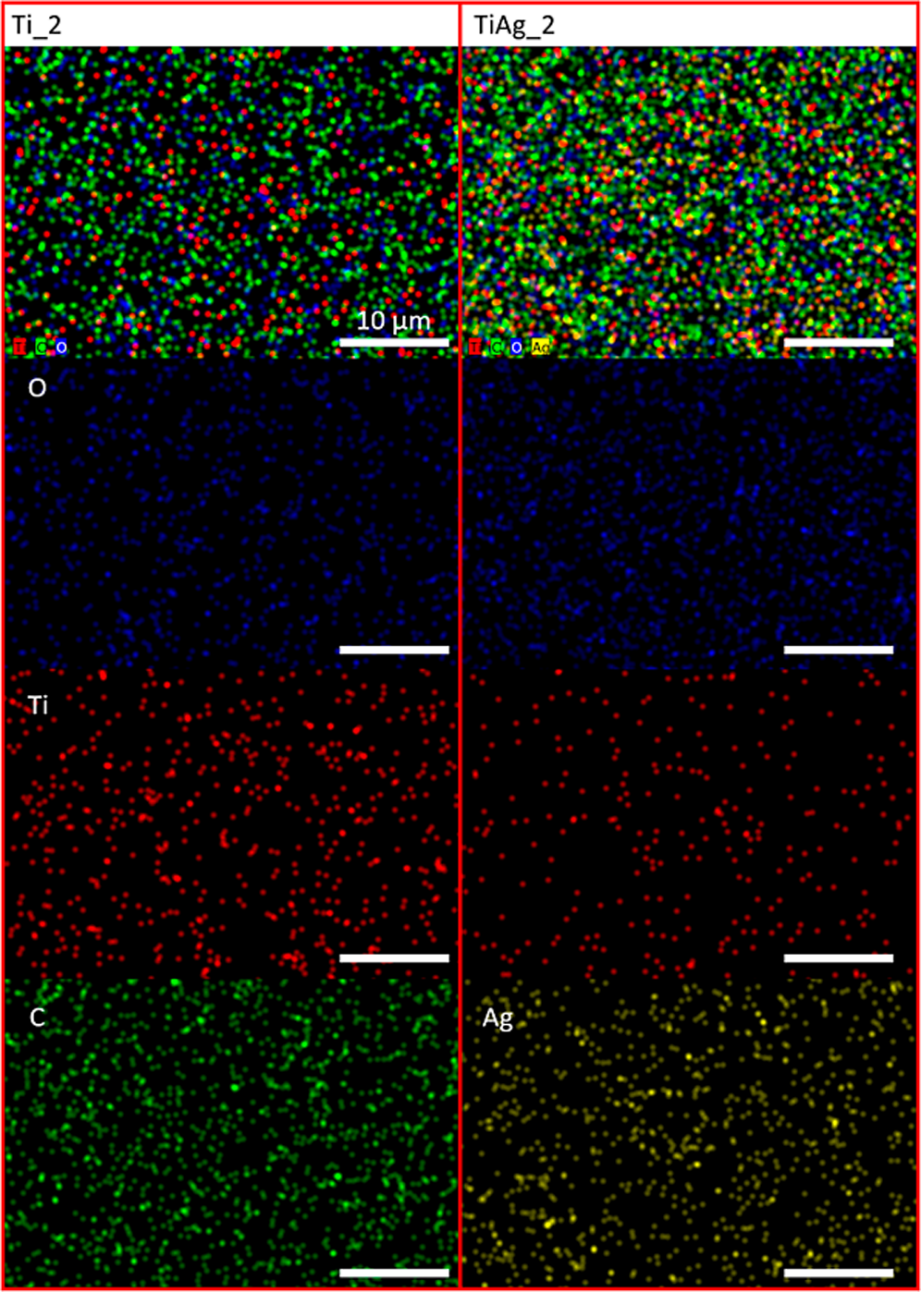
EDS elemental mapping of Ti_2 (left panel) and TiAg_2 (right panel) films showing the spatial distribution of O, Ti, C, and Ag.

Table 1 presents the elemental compositions determined by EDS. The EDS analysis reveals a positive correlation between increased metal doping and a higher oxygen content in the DLC films. This trend is likely associated with two factors: (i) structural disruption of the DLC matrix due to metal incorporation, resulting in a more amorphous network that facilitates oxygen uptake, and (ii) the increased chemical reactivity of the doped metals toward oxygen. The increase of the Ti content in the DLC films from 4.0 to 6.0 at. % enhanced the oxygen concentration from 11.2 to 15.5 at. % (Table 1).

**Table 1:** Deposition Parameters and the Chemical Composition of Ti-DLC and Ti/Ag-DLC Films

Sample	Deposition parameter	EDS	XPS								
	Shutter opening (mm)	C (at.%)	O (at. %)	Ti (at. %)	Ag (at. %)	C (at. %)	O (at. %)	Ti (at. %)	Ag (at. %)	Ratio sp^2^/sp^3^	sp^3^/(sp^2^+sp^3^)
DLC	-	89.9	10.1	-	-	82.6	17.4	-	-	1.09	0.48
Ti_1	16	84.8	11.2	4.0	-	77.4	22.3	0.3	-	1.12	0.47
Ti_2	32	81.9	12.9	5.2	-	77.4	21.8	0.8	-	1.11	0.47
Ti_3	-	78.5	15.5	6.0	-	75.5	22.7	1.8	-	1.20	0.45
TiAg_1	16	89.6	7.4	1.7	1.3	82.6	16.4	0.4	0.6	1.09	0.48
TiAg_2	32	84.1	9.4	4.0	2.5	76.8	20.0	0.9	2.3	1.18	0.46
TiAg_3	-	74.9	15.2	6.4	3.5	70.6	22.5	1.0	5.9	1.39	0.42

The codoped Ti/Ag-DLC films had a lower content of oxygen. The enhancement in the Ti/Ag metal contents resulted in the oxygen concentration increasing from 7.4 to 15.2 at. %. For TiAg_1 and TiAg_2 films, the oxygen fraction decreases compared to the DLC film, suggesting that the silver can suppress oxidation when present at low concentrations. Most doped DLC films exhibit gradual variations in elemental composition; however, a pronounced change is observed between codoped TiAg_2 and TiAg_3 films. This abrupt shift suggests that beyond a critical doping threshold, silver no longer effectively suppresses oxygen incorporation. The corresponding increase in the oxygen content likely reflects a significant alteration in the DLC structure, indicating a transition toward a more disordered structure. Titanium’s high oxygen affinity means that upon air exposure, Ti-doped DLC surfaces rapidly oxidize. XPS consistently reveals Ti 2p doublets characteristic of TiO_2_/TiO_*x*_ and a concomitant increase in O 1s Ti–O contributions, while metallic Ti signals are absent or negligible.^18,34^

The surface composition of the DLC film, as analyzed by X-ray photoelectron spectroscopy (XPS), is presented in Table 1. Given that XPS probes only the upper few nanometers of a material, the relatively high oxygen levels observed can be attributed to enhanced surface oxidation upon exposure to ambient air. All three Ti-doped DLC films exhibited comparable oxygen contents, averaging around 22 at. %. In contrast, the Ti/Ag codoped DLC films showed a broader range of oxygen concentrations, from 16.4 to 22.5 at. %, which tended to increase with higher total dopant loading. However, only the TiAg_1 film demonstrated a lower oxygen content than the undoped DLC. The Ti content remained nearly unchanged between Ti-DLC and corresponding Ti/Ag codoped DLC films at lower doping levels, with Ti_1 and TiAg_1 films containing 0.3 and 0.4 at. % and Ti_2 and TiAg_2 films showing 0.8 and 0.9 at. %, respectively. At the highest doping level, the surface composition became increasingly dominated by Ag, while Ti plateaued. The Ti_3 film contained 1.8 at. % Ti, whereas the TiAg_3 film had only 1.0 at. %. The Ag signal increased almost 10-fold, from 0.6 at.% in the TiAg_1 film to 5.9 at. % in the TiAg_3 film.

XPS revealed that the surface is significantly enriched with Ag. Cloutier et al. studied distribution and clustering of silver in Ag-DLC.^35^ Their XPS analysis of biased Ag-DLC coatings (from −50 to −150 V) revealed pronounced surface segregation of silver, consistent with its low solubility in carbon matrices. The authors attributed this behavior to the ion subimplantation mechanism characteristic of DLC growth, wherein hyperthermal carbon ions are implanted into subsurface sites, densifying the film and enhancing sp^3^ bonding. In contrast, heavier Ag atoms lack sufficient energy to penetrate deeply and instead remain near the surface, where local heating further promotes their diffusion and segregation.

XPS scan spectra of the signals of C 1s, –O 1s, -Ti 2p, and Ag 3d are presented in Figure 2. Figure 2a shows the XPS spectrum of deconvolution of C 1s for undoped DLC. Four component positions were identified at 283.7, 284.6, 286.7, and 287.9 eV, which correspond to C=C (sp^2^), C–C (sp^3^), C–O/C–OH, and C=O, respectively.^15,16,36^ The fitting procedure was stabilized by applying literature-supported binding energy ranges and by constraining the chemical shift between the sp^2^ and sp^3^ components to ∼0.9 eV.^36,37^ As seen in Figure 2b,c, the C 1s peak of all DLC films shows a similar curve shape toward the low energy region. If metal–carbon bonds are formed, the broadening or the appearance of a new peak is typically observed in the C 1s region between 283 and 281 eV, indicating the presence of metal–carbide (i.e., Ti–C) bonds.^16^ In this case, no such features were detected, suggesting that Ti–C bond formation and carbide development did not occur in the Ti-DLC and Ti/Ag-DLC films. This absence can be attributed to the relatively low deposition temperature (220–230 °C), which is insufficient to promote titanium carbide formation.

**Figure 2:**
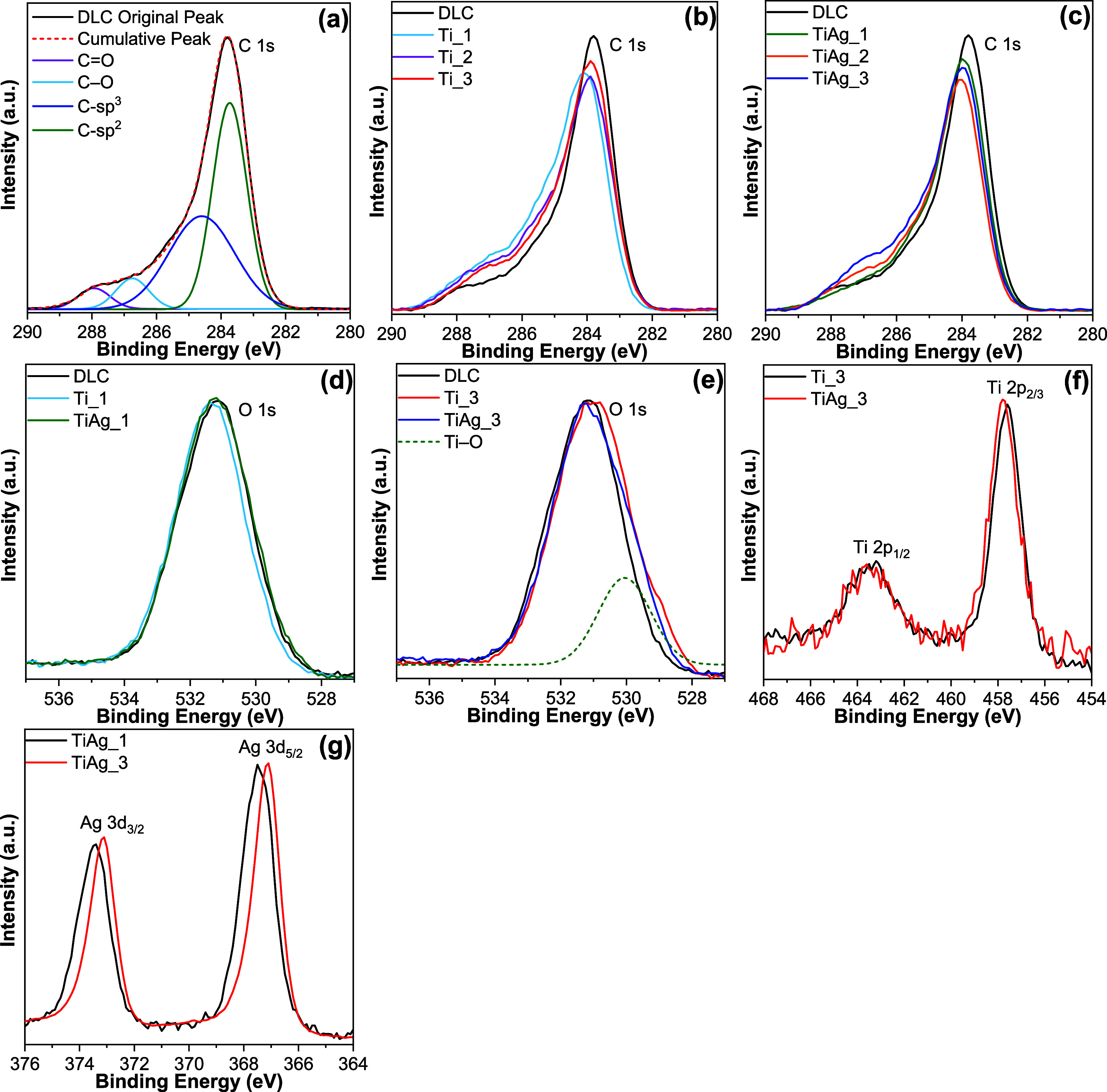
High-resolution XPS spectra of the DLC series: (a) C 1s spectrum of the undoped film with peak deconvolution; (b) C 1s spectra comparing undoped DLC and Ti-DLC; (c) C 1s spectra comparing undoped DLC and Ti/Ag-DLC; (d) O 1s spectra at the lowest dopant loadings, contrasting Ti-only and Ti/Ag codoped films; (e) O 1s spectra at the highest dopant loadings for the same comparison; (f) Ti 2p spectra of Ti_3 and TiAg_3 films; and (g) Ag 3d spectra of TiAg_1 and TiAg_3 films.

Figure 2d,e shows the shift of the O 1s spectra with the lowest and the highest metal dopant contents. With the introduction of metal dopants, the peak broadens to a lower energy region, which is shown as a deconvoluted peak at 530.0 eV. The peak at 530.0 eV has been widely attributed to the Ti–O bond or TiO_2_.^18,38,39^ This matches well with the difference in Ti content in the Ti_3 and TiAg_3 films. Písařík et al. reported that the presence of Ag can also shift the O 1s peak toward lower energy, and the authors attributed the deconvoluted peak at 530.7 eV to O=Ag and O=C bonds.^36^ Figure 2f shows overlaid peaks of the Ti 2p state for Ti_3 and TiAg_3 film spectra. Two separate peaks can be seen at around 457.7 and 463.5 eV, which are commonly attributed to the existence of TiO_2_ (Ti^4+^ state) in Ti-DLC films.^16,18,40^ Other authors have reported XPS spectra of Ti-doped DLC that contain a Ti 2p doublet centered at ∼455 eV (2p_3/2_) and ∼461 eV (2p_1/2_); these binding energies are characteristic of Ti–C bonding.^41–43^ But these peaks of Ti–C at around 455 and 461 eV are not visible in the spectra of Ti_3 and TiAg_3 films. The XPS results clearly demonstrate that the Ti bonded with the oxygen and created mainly TiO_2_ bonds in both Ti-DLC and Ti/Ag-DLC films. High-resolution Ag 3d spectra (Figure 2g) collected from the Ti/Ag doped DLC films display characteristic doublets at ∼367 eV (Ag 3d_5_/_2_) and ∼373 eV (Ag 3d_3_/_2_). Cloutier et al. summarized previous studies, showing that oxidation can shift the Ag 3d_5_/_2_ peak toward lower binding energy, with Ag_2_O and AgO exhibiting binding energies of 367.8 and 367.4 eV, respectively, which are lower than that of metallic silver (368.4 eV).^44^ Thus, surface silver nanoclusters were at least partially oxidized. Figure 2g shows that the Ag 3d_3/2_ and 3d_5/2_ peaks become narrower and shift slightly to lower values as silver content increases, indicating reduced bonding disorder and the formation of larger Ag clusters.^36^

The sp^2^/sp^3^ and sp^3^/(sp^2^ + sp^3^) ratios extracted from the high-resolution C 1s deconvolution (Table 1) show that metal doping progressively shifts the carbon network toward a more graphitic configuration. At the lowest dopant levels (Ti_1, Ti_2, TiAg_1; ≤ ∼ 1.0 at. % total metal), the change is modest: the sp^2^/sp^3^ ratio rises by only up to 3% relative to undoped DLC and the sp^3^ fraction remains from 0.47 to 0.48. A clearer shift appears in Ti_3 and TiAg_2 (1.8 and 3.2 at. % total metal content, respectively), where the sp^2^/sp^3^ ratio increases by up to 10% and the sp^3^ fraction falls to ∼0.45. When the total Ti and Ag content reaches 6.9 at. % in the TiAg_3 film, the effect becomes pronounced: the sp^2^/sp^3^ ratio is 27.5% higher than the baseline and the sp^3^ fraction drops to 0.42. These results indicate that the extent of the metal-induced shift in hybridization scales nonlinearly with overall dopant concentration. Towobola et al. showed that Ag incorporation progressively converts sp^3^ to sp^2^ bonding in DLC.^45^ The change is minor below ∼5 at. % Ag but becomes pronounced once the Ag content reaches 7.1 at. %, as reflected by both XPS (diminished sp^3^ C–C peak) and a sharp rise in the Raman *I*_D_/*I*_G_ ratio.

To gain insight into the structural evolution of DLC films, Raman spectroscopy was employed as it provides crucial information on the phase composition. Figure 3 displays the Raman spectra of the DLC films deposited at varied shutter openings for the Ti and Ti/Ag targets. All spectra exhibited a broad, asymmetric peak in the range of 1100–1750 cm^–1^, characteristic of DLC films.^46^ This envelope consists of two overlapping peaks: the D peak (around 1350–1400 cm^–1^) and the G peak (around 1550–1580 cm^–1^).^47^

**Figure 3:**
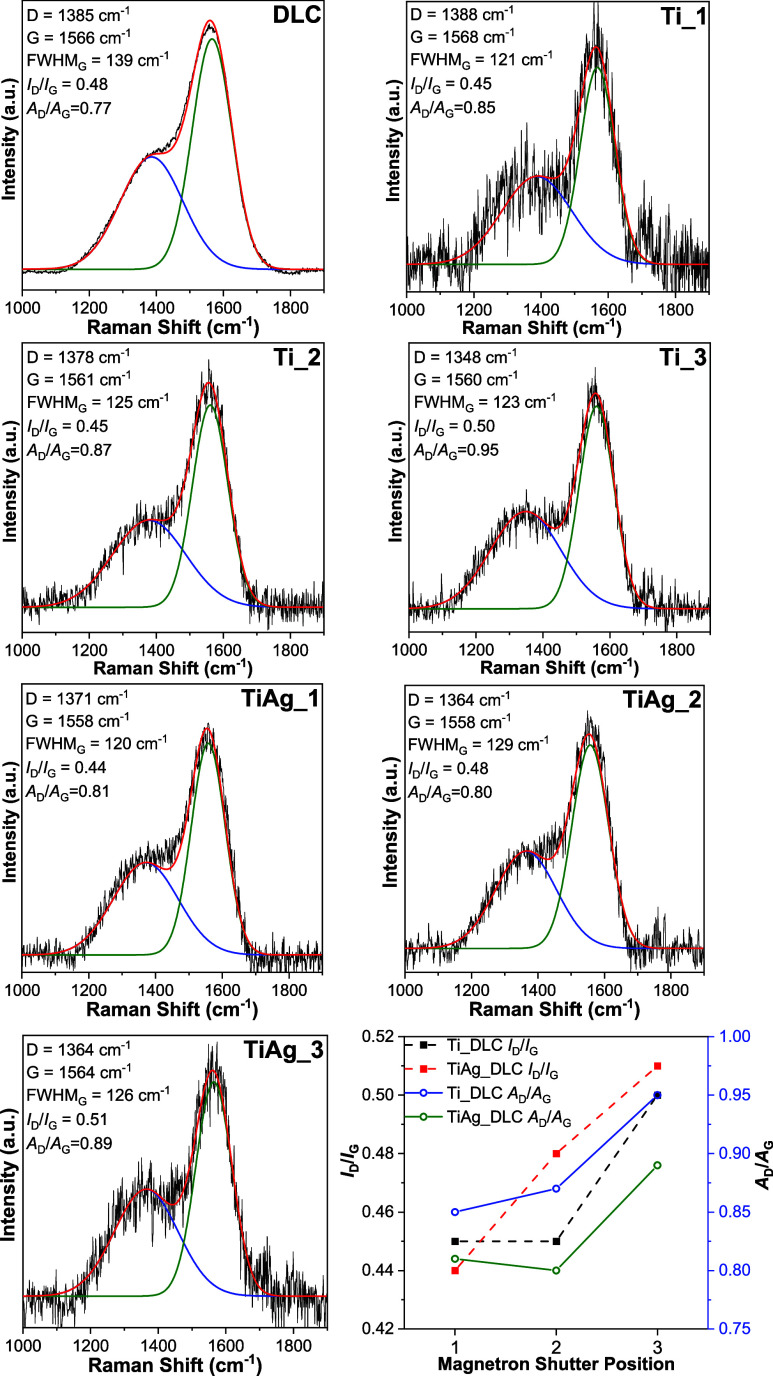
Raman spectra of the undoped DLC, Ti-DLC, and Ti/Ag-DLC films, together with their characteristic spectral parameters plotted as a function of the magnetron shutter position.

The G peak of the undoped DLC film was observed at 1566 cm^–1^, and the introduction of metallic dopants caused only minor variations in its position, which remained within a range of 1558–1568 cm^–1^. Although the full width at half-maximum (fwhm) of the G peak decreased in all doped DLC films, no clear trend could be established in relation to the metal content (Figure 3). In contrast, the D peak exhibited a noticeable shift toward lower wavenumbers with increasing metal concentration. The D peak position of the undoped DLC film was at 1385 cm^–1^ and slightly moved to 1388 cm^–1^ with the addition of the lowest amount of Ti. The further increase of the Ti concentration reduced the D band position to 1378 cm^–1^ and 1348 cm^–1^ for the Ti_2 and Ti_3 films, respectively. The codoping of the DLC film with the lowest amount of Ti/Ag reduced the D peak position down to 1371 cm^–1^. Meanwhile, the D band position of the TiAg_2 and TiAg_3 films was fixed at 1364 cm^–1^. The increase in the Ti or Ti/Ag concentration promoted the growth of larger sp^2^ clusters. The larger size C=C sp^2^ domains have more delocalized electrons and a lower vibrational frequency; as a result, the D peak is shifted to lower wavenumbers.^48^

The sp^3^ content was estimated using the Ferrari–Robertson model^49,50^ by correlating the G-peak position, *I*_D_/*I*_G_ ratio, and fwhm_G_. Across all films, the derived sp^3^ fraction lies in the range ∼27–35%, consistent with Stage-2 amorphous carbon (DLC). It should be noted that the Raman spectral parameters primarily reflect the structure and clustering of the sp^2^ phase, not the absolute sp^3^ C–C fraction. Still, doping with Ti and Ti/Ag does not significantly reduce the sp^3^ network, which aligns with the XPS-derived bonding trends.

Metal-doped Ti-DLC and Ti/Ag-DLC films showed a trend where the *I*_D_/*I*_G_ ratio increased with metal dopant content. In addition, increasing metal content is also accompanied by increasing oxygen content. It has been reported that *I*_D_/*I*_G_ ratio increases with disorder and formation of larger sp^2^ clusters, reflecting greater graphitization in the DLC films.^51^ According to Tai et al.,^52^ an increase in *I*_D_/*I*_G_ ratio correlates with reduced sp^3^ content, which the authors supported by independent XPS measurements. These trends are consistent with the recent literature, including studies by Rao et al.^53^ and Samiee et al.,^54^ who demonstrated that *I*_D_/*I*_G_ ratio increases with greater disorder and graphitization in DLC films. At the same time, the area ratio (*A*_D_/*A*_G_), derived from the integrated area under the deconvoluted D and G peaks, showed a similar increasing trend with higher metal doping levels. Dovydaitis et al. demonstrated that the *A*_D_/*A*_G_ ratio increases with rising chromium content in Cr-doped DLC films, reflecting Cr-induced graphitization and the formation of larger sp^2^ ring clusters.^55^

### 3.2 Morphology and Surface Properties

AFM micrographs (Figure 4) reveal that all films retain the smooth character typical of sputtered DLC, but subtle textural changes emerge with metal incorporation. In the Ti-doped series, the surface displays a fine distribution that becomes progressively more concentrated as the dopant level increases. When Ag is introduced together with Ti, the sputtered clusters are not only more numerous but also appear larger, giving the codoped films a more pronounced granular relief compared with their Ti-only counterparts.

**Figure 4:**
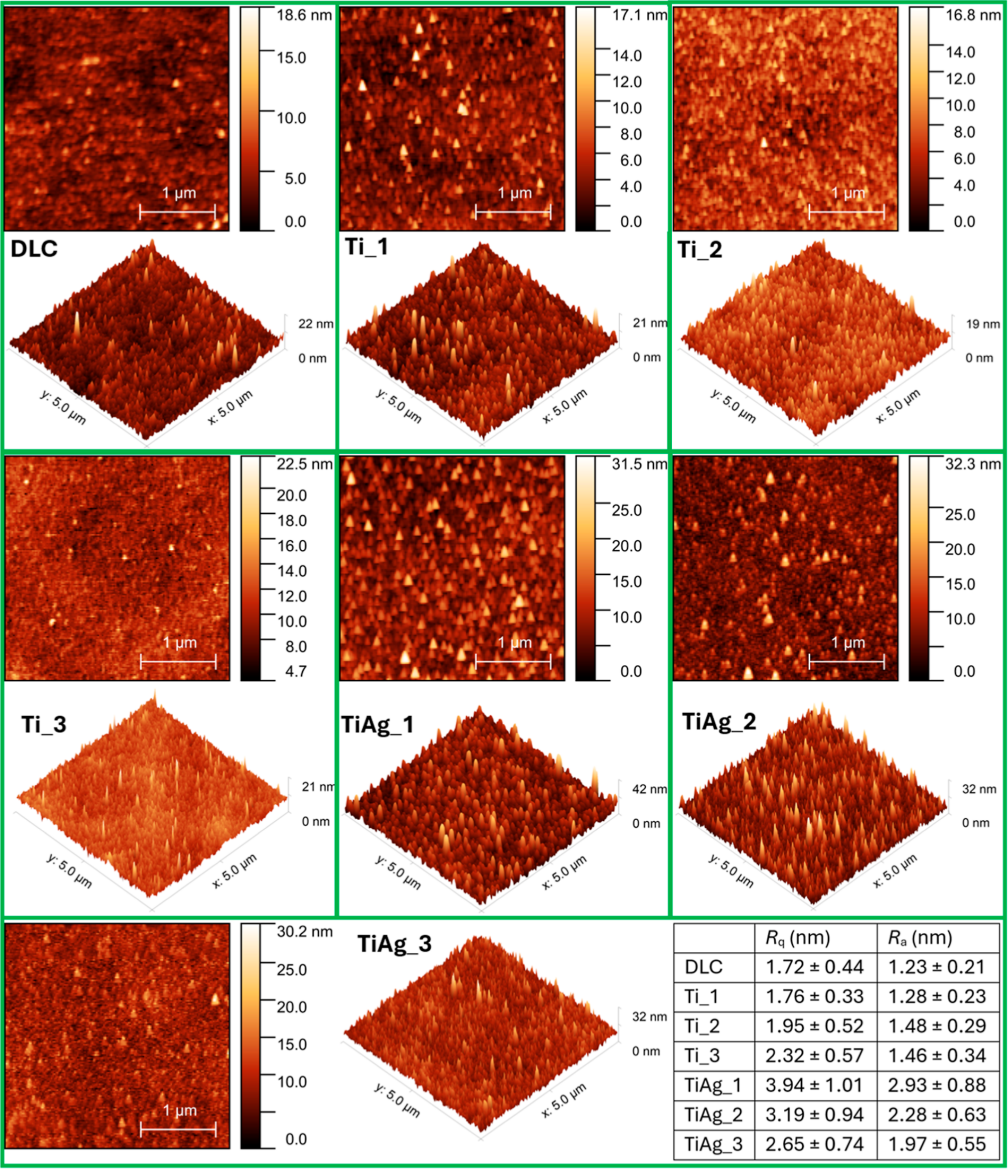
AFM surface morphology micrographs 2D (3 × 3 μm^2^) and 3D (5 × 5 μm^2^) for Ti-DLC and Ti/Ag-DLC films at various dopant levels. The accompanying table lists *R*_q_ (root mean square roughness) and *R*_a_ (arithmetic average roughness) for each film.

*R*_q_ (Root Mean Square Roughness) and *R*_a_ (Arithmetic Average Roughness) are also provided in Figure 4. Increasing titanium (Ti_1 to Ti_3) content introduces a denser field of up to 20 nm high mounds in the Ti-DLC films, but the roughness remains low; *R*_q_ stays within the range 1.8–2.3 nm. These results are consistent with the article by Shen et al., which reported Ti-DLC morphology with mounds of up to 20 nm and the *R*_q_ range of 1.1–2.4 nm but a Ti content of up to 23 at. %.^56^ Zhang et al. reported that raising the Ti content from ∼4.4 to 27.0 at. % increased the *R*_a_ of Ti-DLC coatings from 1.42 to 2.64 nm.^48^ The authors demonstrated that Ti doping lowers the migration energy of carbon atoms, which in turn yields Ti-DLC coatings with a slightly rougher surface. They attributed this roughness increase to the emergence of Ti–C nanoclusters combined with the Ti-induced reduction in the carbon-atom migration energy.

Co-doping with silver and titanium produces a different evolution of the surface. At the lowest Ti/Ag codoped content in the DLC film (TiAg_1), the surface is filled with discrete ∼50 nm in diameter islands/mounds, giving the highest roughness in the series. When the total metal atom flux was increased, these islands began to coalesce, the gaps between them progressively filled, and both *R*_q_ and *R*_a_ decreased from 3.9 to 2.7 nm and 2.9 to 2.0 nm, respectively. These *R*_a_ values match with the data observed in the article by Jing et al., which showed a similar tendency of surface roughness shift with increasing Ag content from 0.6 to 10.0 at.% *R*_a_ fluctuated between 2.2 and 3.5 nm.^11^ It should be noted that there is a large disparity in sputter yields between Ag and Ti, with Ag being deposited about 5-fold higher, according to tabulated sputter rate measurements.^57,58^ Thus, the early silver supersaturation promotes rapid nucleation and an initial roughness spike. By contrast, the lower-flux Ti-only process yields uniformly dispersed Ti atoms, resulting in a slight roughness rise within the Ti-DLC series.

Figure 5a,b shows the nanofriction force versus the applied normal load. A similar range of values and trend was observed for metal-doped DLC films. Notably, the slope of each curve gives the nanoscale coefficient of friction, which is listed in the figure insets. For undoped DLC, the friction coefficient is 1.14. Introducing a modest amount of Ti halves this value: Ti_1 and Ti_2 show friction coefficients of 0.57 and 0.66, respectively. When the Ti content is increased further (Ti_3), the trend reverses, and the coefficient of friction reaches 1.26 and becomes higher than that of undoped DLC. Guo et al. investigated Ti-doped DLC coatings under dry sliding and observed that the coefficient of friction increases as Ti content increases.^59^ According to the authors, a small Ti content promotes slight graphitization and the formation of a transfer film, but once the surface is saturated, Ti-based hard phases (e.g., TiC/TiO_*x*_) dominate the contact and drive the friction higher.

**Figure 5:**
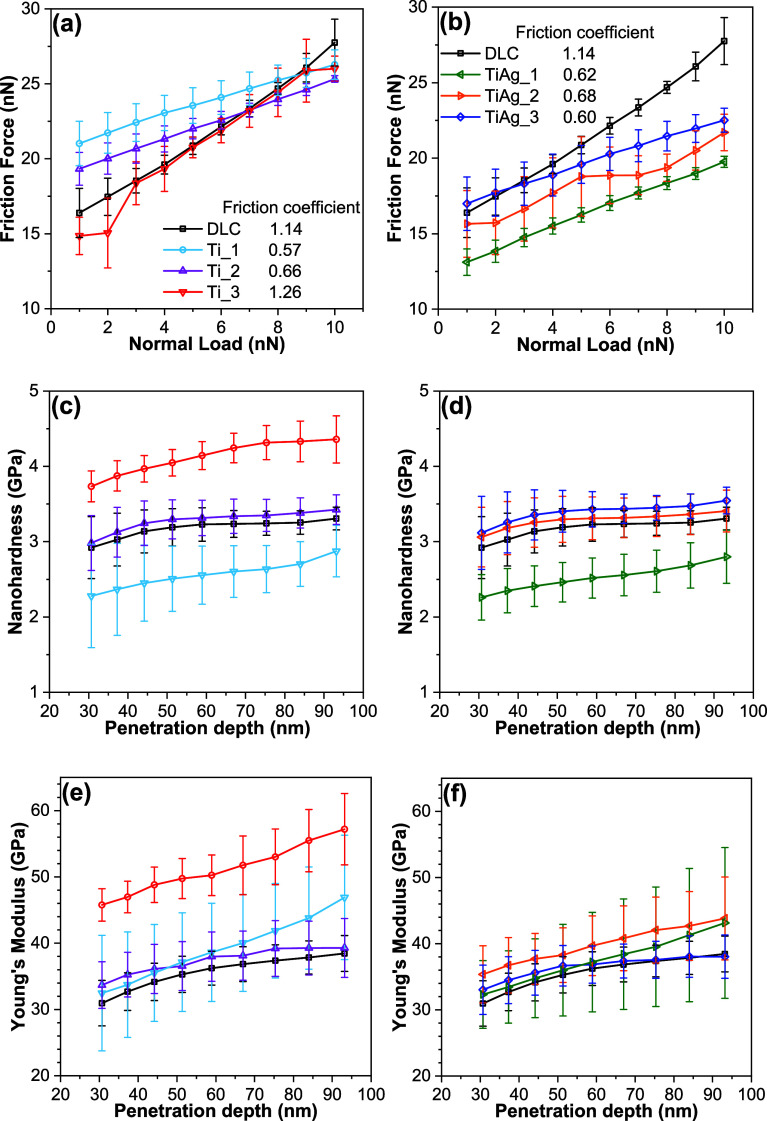
Dependence of lateral friction force on applied normal load for (a) Ti-DLC and (b) Ti/Ag-DLC; the corresponding friction coefficients are given in the insets. Nanoindentation results as a function of penetration depth: nanohardness for (c) Ti-DLC and (d) Ti/Ag-DLC and Young’s modulus for (e) Ti-DLC and (f) Ti/Ag-DLC films.

The literature indicates that Ag nanoinclusions are mechanically soft and rapidly enrich the sliding interface, providing an immediate but load-sensitive shear plane.^11^ Jing et al. showed that once the silver content in Ag-DLC exceeds about 3.2 at. %, both nanohardness and compressive stress fall sharply.^11^ This mechanical softening accelerates wear and undermines the structural integrity of the film. Kolodziejczyk et al. studied the nanotribology of hydrogenated Ag-DLC and reported coefficient of friction values ranging from around 0.5 to 0.8 with Ag content from around 5 to 15 at. %.^5^ Our Ti/Ag-DLC films show similar friction coefficient values fluctuating between 0.60 and 0.68, which yielded a 1.7- to 1.9-fold reduction compared to undoped DLC.

As the surface gets rougher, friction increases because more asperities come into contact, enlarging the real contact area and shifting the mechanism from mainly adhesive to more abrasive wear.^33^ The wear rate, however, still depends on the film’s own mechanical properties (Table 2): harder, stiffer (higher modulus) DLC resists material removal but often shows a higher friction force under the same load.^7^ In our samples, this could partly explain why friction rises again at higher Ti content for Ti-DLC films.

**Table 2:** Data on Nanoindentation Test Parameters and Their Corresponding Ratios

Sample	Hardness (*H*) (GPa)	Young’s modulus (*E*) (GPa)	*H*/*E*	*H* ^3^/*E* ^2^ (GPa)
DLC	3.17 ± 0.24	35.5 ± 2.7	0.089	0.025
Ti_1	2.55 ± 0.43	38.9 ± 7.8	0.066	0.011
Ti_2	3.27 ± 0.25	37.3 ± 3.8	0.088	0.025
Ti_3	4.11 ± 0.21	51.0 ± 3.6	0.081	0.027
TiAg_1	2.52 ± 0.28	37.4 ± 7.7	0.067	0.011
TiAg_2	3.28 ± 0.31	39.7 ± 4.7	0.083	0.022
TiAg_3	3.39 ± 0.27	36.4 ± 3.1	0.093	0.029

The results of the nanoindentation measurements are presented in Figure 5c–f, while the analysis of the data from the curves for the doped DLC films is provided in Table 2. The DLC film exhibited a nanohardness of 3.17 GPa and a Young’s modulus of 35.5 GPa. The Ti_1, Ti_3, and TiAg_1 films deviate noticeably from undoped DLC, whereas the other samples lie within the experimental scatter. Notably, introducing small amounts of Ti or codoped Ti/Ag resulted in a drop of nanohardness, while Young’s modulus values remained within the margin of error compared to undoped DLC. Both Ti_1 and TiAg_1 show very similar values for nanohardness of 2.55 and 2.52 GPa, respectively. Wei et al. reported that the introduction of 2.7 at. % Ti led to a decrease in both nanohardness and Young’s modulus when compared to neat DLC.^60^ But at the same time, introducing 2.7 to 7.7 at. % led to progressive internal compressive stress reduction. Zhang et al. reported a similar tendency with Ti-doped DLC and also observed that above a Ti content of 10 at. %, the hardness and Young modulus stated to increase.^48^ The authors attributed the initial step to the uniform dissolution of Ti atoms within the amorphous carbon matrix, which led to the formation of small amounts of nanocrystalline carbides. Sun et al. reviewed the effects of doping on DLC films, noting that most element dopants, including Ti, generally cause a decrease in hardness.^61^ In the current study, no carbides were detected in the XPS spectra, while pronounced peaks corresponding to titanium oxides were observed instead. Although the Ti_3 film exhibited enhanced nanohardness relative to undoped DLC, the measured values are still consistent with oxygen-rich DLC and remain lower than those typically observed when TiC phases are present.^59^

Ag-doped DLC films as reported in the literature become softer as the silver content increases beyond a low threshold.^36,62^ The reduction in hardness is more severe than that with Ti because Ag does not contribute to any hard phase; it only promotes graphitization. The values of nanohardness (*H*) and Young’s modulus (*E*) are strongly linked together and often examined in ratios *H*/*E* and *H* ^3^/*E* ^2^. Higher H/E ratios have been shown to correlate with improved wear resistance, i.e., enhanced ability to absorb and dissipate energy during mechanical contact,^63^ while higher *H* ^3^/*E* ^2^ has been correlated with better resistance to plastic deformation, which can prevent wear and cracking.^63^ Chen et al. cautioned about dependency on these ratios for analysis as they do not fully reflect complex structure/properties relationships in nanolayer and nanocomposite coatings.^64^ When the ratios are examined, Ti_1 and TiAg_1 films exhibit a significant drop in both parameters. In contrast, the *H*/*E* (0.093) and *H* ^3^/*E* ^2^ (0.029) ratios favor TiAg_3, which is interesting as the absolute values favor Ti_3. Taken together, these observations suggest that moderate dopant concentrations rather than low concentrations yield better mechanical performance.

The wettability of the doped DLC films was assessed through contact angle (CA) measurements. Based on these measurements (summarized in Table 3), the total surface free energy (γ_tot_) was estimated using the Owens, Wendt, Rabel, and Kaelble (OWRK) method, which divides the surface energy into its dispersive (γ_d_) and polar (γ_p_) components. The undoped DLC exhibited a water contact angle of 65.2°, aligning with the commonly reported range of ∼65–80° for DLC films.^65,66^ Incorporation of Ti produced a modest increase in the water contact angle to around 69° for all Ti-DLC films. In contrast, the Ti/Ag codoped DLC films displayed an initial decrease (59.9°) followed by recovery and increase for TiAg_2 and TiAg_3 films, respectively. The contact angle can be influenced by both intrinsic surface chemistry, e.g., changes in dispersive/polar surface energy components due to dopant-induced bonding and oxidation states, and nanoscale topography. Thus, it is important to decouple these effects when interpreting the observed trend. The DLC films examined in this study exhibit a very low roughness (*R*_q_ = 1.72–3.94 nm). According to the literature, roughness at this scale does not measurably influence surface wettability.^67,68^

**Table 3:** Contact Angle Values and Surface Free Energy Components of Doped DLC Films

sample	contact angle			surface energy–OWRK		
	θ_W_ (°)	θ_DM_ (°)	θ_EG_ (°)	γ_tot_ (mN/m)	γ_d_ (mN/m)	γ_p_ (mN/m)
DLC	65.2 ± 2.4	45.6 ± 2.8	50.1 ± 5.4	43.5	35.0	8.5
Ti_1	69.3 ± 3.0	55.0 ± 1.9	54.0 ± 1.5	38.3	29.2	9.1
Ti_2	69.2 ± 1.6	52.8 ± 2.7	52.7 ± 1.5	39.1	30.4	8.7
Ti_3	68.8 ± 1.7	48.0 ± 1.7	50.4 ± 1.8	41.0	33.0	8.0
TiAg_1	59.9 ± 2.1	45.5 ± 2.5	49.2 ± 2.7	44.2	32.6	11.6
TiAg_2	64.7 ± 1.3	52.3 ± 2.2	50.4 ± 1.0	41.1	30.3	10.8
TiAg_3	68.5 ± 2.4	49.9 ± 2.5	48.4 ± 1.7	41.0	32.3	8.7

Sun et al. reviewed doping effects on the DLC coatings and reported that doping generally raises the sp^2^ carbon fraction in DLC films, and a higher sp^2^-rich surface can lower surface free energy (fewer dangling bonds) and thus increase hydrophobicity.^61^ However, the authors noted that the wettability does not vary monotonically with the Raman *I*_D_/*I*_G_ ratio; water CA and surface energy exhibit a nonmonotonic dependence because wettability also depends on the type, size, and distortion of sp^2^ clusters (rings vs chains, degree of disorder), not merely the overall sp^2^/sp^3^ ratio. Travnickova et al. investigated DLC films doped with 0.4, 2.1, 3.7, 6.6, and 12.8 at. % Ti and observed a gradual rise in the water CA from 76.8° (undoped) to 77.7°, 78.0°, 78.4°, 80.5° and 86.3°, respectively.^65^ Because the roughness (*R*_a_) remained extremely low, between 0.18 and 0.36 nm across all compositions, the progressive increase in contact angle can be attributed primarily to Ti-induced surface chemical modifications. MubarakAli et al. examined ta-C films doped with 0.63, 1.34, and 2.01 at. % Ag, finding only minor changes in roughness, *R*_a_ = 1.4, 1.3, and 1.7 nm, respectively.^66^ Nevertheless, the water CA increased systematically from 64.2° for undoped ta-C to 70.80°, 76.48°, and 79.43° with increasing Ag content. This hydrophobic shift is therefore attributed primarily to Ag incorporation, i.e., to (i) a reduction in the density of polar carbon surface sites and (ii) the onset of nanocluster-induced local graphitization.

The literature data indicate that the Ti-DLC film results are within the expected range of changes as all three compositions saw an increase in oxygen content by about 5 at. % (Table 1). All three Ti-DLC films showed similar water CA values around 69°, which can be explained with relatively similar sp^2^/sp^3^ ratios and data scatter (errors of up to 3°). In the Ti/Ag codoped DLC films, as total dopant concentration increases, the surface oxygen concentration (XPS) rises from 16.4 to 22.5 at. % and Ag content increases from 0.6 to 5.9 at. % (XPS). TiAg_1 exhibits an sp^2^/sp^3^ ratio essentially identical with that of the undoped DLC and a comparatively lower oxygen level. This combination limited graphitization and shifted oxidized states of elements, which can account for its reduced water CA. It should be noted that the sp^2^-rich surfaces with π electrons are less wettable.^69^ As the Ti and Ag content increases, local graphitic clustering is promoted, and the surface oxygen concentration correspondingly increases, thus contributing to changes observed for TiAg_2 and TiAg_3. The OWRK analysis reveals a decrease in the total surface free energy arising from concurrent reductions in the dispersive components. At low dopant levels, the polar surface-energy component rises slightly, but it declines steadily in both Ti-DLC and Ti/Ag-DLC films as the dopant concentration increases. This trend is consistent with dopant-induced graphitization and enhanced sp^2^ clustering, which together suppress γ_p_ and γ_d_, thereby lowering γ_tot_.

The investigations demonstrated that the doping of DLC films with Ti reduced the friction coefficient from 1.14 to 0.57 and 0.66 when the Ti concentrations were 0.3 at. % (4.0 at. % by EDS) and 0.8 at. % (5.2 at. % by EDS), respectively. The nanoscale friction coefficient was minimized when the surface roughness remained similar to that of the DLC film. An addition of Ti led to the formation of Ti–O bonds, and only a slight reduction in the sp^3^ C–C bonds fraction was observed. Despite the increase in the oxygen concentration in the Ti-DLC films, the total surface energy was reduced due to preferential oxygen bonding with Ti instead of the formation of C–O and C=O sites. However, the friction coefficient was increased up to 1.26 due to elevated surface roughness, the sp^2^ C=C site fraction, and the highest oxygen content in the Ti-DLC films when the Ti concentration was 1.8 at. % (6.0 at. % by EDS). The combination of these parameters resulted in the highest surface energy value and an increase in the friction coefficient of the Ti_3 DLC film.

The codoping of the DLC films resulted in increased surface roughness. However, the friction coefficient of the Ti/Ag-DLC films remained in the range of 0.60–0.68. Typically, the reduction of surface energy resulted in lower friction coefficient values at the nanoscale level.^5^ The total surface energy of the Ti/Ag-DLC film with the lowest Ag concentration was the highest. However, the friction coefficient remained at 0.62. The oxygen content in the Ti/Ag codoped DLC films was lower than that of DLC films when Ag content was 0.6 and 2.3 at. %. It is likely that the lower oxygen content results in a lower concentration of Ti–O and C–O bonds, which compensates for the increased surface roughness and surface energy of codoped DLC films. Meanwhile, the DLC film codoped with the highest concentration of the Ag and Ti demonstrated the lowest surface energy and roughness values, leading to the lowest friction value of 0.60, despite the increased oxygen content and the highest fraction of the sp^2^ C=C sites. The study has shown that to reduce the friction coefficient of Ti-DLC and Ti/Ag-DLC films, a combination of several components is important: surface roughness, oxygen concentration, sp^3^/sp^2^ bond ratio, Ti–O, C–O, and C=O bond concentrations, and surface energy.

Collectively, the results indicate that nanoscale friction is minimized under conditions where dopants induce modest graphitic clustering, restrained Ti-mediated oxidation, reduced dispersive surface energy contributions, and moderate mechanical softening of the near-surface carbon network. These conditions are satisfied with the lowest and moderate Ti concentrations and all used Ti/Ag codoping levels of DLC films.

## 4 Conclusions

In this study, non-hydrogenated DLC films doped with Ti or codoped with Ti/Ag were prepared by magnetron sputtering, and their surface and tribological properties were systematically evaluated. XPS and Raman spectroscopies indicated that both Ti doping and codoping with Ti/Ag induced minimal graphitization, reflected by almost no increased sp^2^/sp^3^ and *I*_D_/*I*_G_ ratios, with only TiAg_3 standing out as an exception. The addition of Ti resulted in only minor increases in surface roughness, whereas Ti/Ag codoping initially caused a notable roughness increase, subsequently smoothing as metal content rose in the DLC films. The presence of Ag significantly affected the surface oxygen content, which was reduced at low to moderate doping levels. At the same time, Ti presence increased oxidation of the surface, but overall oxygen content remained independent of Ti content in Ti-DLC films.

Doping with Ti or Ti/Ag reduced the coefficient of friction by approximately 1.7- to 2-fold relative to undoped DLC. The only exception was the highest Ti loading (Ti_3), which showed a slight increase above the reference value. Nanoindentation shows that small additions of Ti or Ti/Ag soften the DLC relative to the undoped film (*H* = 3.17 GPa). With further Ti incorporation, the hardness recovers and surpasses the baseline, increasing from 2.55 at 0.3 at. % Ti to 4.11 GPa at 1.8 at. % Ti. A similar, though less pronounced, increase is observed for the codoped series, where nanohardness rises from 2.52 to 3.39 GPa as the total metal content increases. Contact angle measurements demonstrated that the Ti doping modestly enhanced surface hydrophobicity, whereas Ti/Ag codoped DLC films showed a complex, nonmonotonic wetting response influenced by competing chemical and topographical effects. Overall, the study demonstrates that carefully balancing Ti and Ag codopants in DLC films enables simultaneous control over hardness, friction coefficient, roughness, and wettability.

## Data Availability

The data supporting the findings of this study are included within the article. Additional data are available from the corresponding authors upon reasonable request.
